# Long-term effects of composted cattle manure on biomass production, nutritive value, and digestibility of dwarf late Napier grass

**DOI:** 10.3389/fpls.2026.1914669

**Published:** 2026-09-15

**Authors:** Ahmad Wadi, Fitriana Akhsan, Basri, Mohammad Mijanur Rahman

**Affiliations:** 1 Animal Feed Technology Study Program, Animal Husbandry, Polytechnic of Agriculture Pangkep State, Pangkep City, Indonesia; 2 Livestock Production Programme, Faculty of Sustainable Agriculture, Universiti Malaysia Sabah, Sandakan, Sabah, Malaysia

**Keywords:** biomass production, crude protein, fiber fraction, *in vitro* digestibility, organic fertilization, sustainable fodder system

## Abstract

Composted cattle manure provides essential nutrients and organic matter that improve soil fertility and support forage production on marginal lands. Dwarf late (DL) Napier grass [*Cenchrus purpureus* (Schumach) Morrone (syn. *Pennisetum purpureum* Schumach)] is a high-yielding tropical forage whose productivity and nutritive value depend heavily on appropriate nutrient management. This study evaluated the long-term effects of composted cattle manure application on the biomass production, nutritive value, fiber composition, and digestibility of DL Napier grass over two consecutive years under a tropical cut-and-carry system. A field experiment was conducted on marginal soils using a randomized complete block design with three replications. Four manure application rates were evaluated: 0.00, 1.38, 2.88, and 4.67 g nitrogen (N) m^−2^ per application. Over both experimental years, manure application linearly enhanced dry matter yield (DMY) and crude protein (CP), while decreasing structural fiber fractions. Annual DMY in the first year increased significantly (p<0.05) from 339.1 g m^-2^ in the control to 3375.5 g m^-2^ under the highest manure rate. Concurrently, mean CP content increased dose-dependently from 9.32 g 100 g^−1^ to 14.65 g 100 g^−1^ in Year 1, whereas neutral detergent fiber (NDF) and acid detergent lignin (ADL) decreased. Forage quality improved with increasing manure application; notably, stem *in vitro* dry matter digestibility (IVDMD) in the second year rose from 71.87 g 100 g^−1^ (control) to 77.93 g 100 g^−1^ at the highest dose during Cutting III. It is concluded that applying composted cattle manure at the highest rate of 4.67 g N^-2^ per application performed best, providing a sustainable strategy to maximize biomass yield and structural tissue quality under marginal tropical conditions.

## 1 Introduction

The use of manure as a soil amendment has gained increasing attention as a sustainable approach to improving soil fertility while reducing dependence on chemical fertilizers. Manure improves soil physical, chemical, and biological properties by increasing organic matter (OM), enhancing nutrient availability, and stimulating microbial activity, thereby supporting plant growth and productivity. These improvements can result in forage yields comparable to those achieved with inorganic fertilizers (Carricondo-Martínez et al., 2022). In addition, the integration of livestock and crop systems promotes efficient nutrient recycling, reduces environmental impacts, and enhances overall farm sustainability (Du et al., 2022).

Previous studies have demonstrated that manure application significantly increases soil OM, total nitrogen (N), and available phosphorus, which are essential for plant growth and development (Wang et al., 2022a). Poultry manure, in particular, enhances microbial biomass and activity, accelerating nutrient cycling and improving soil fertility (Mahmoud et al., 2023). Partial replacement of inorganic fertilizers with manure has also been reported to improve forage productivity through enhanced soil organic carbon and N availability (Ning et al., 2022). Furthermore, manure application can increase crude protein (CP) concentration and improve soil structure and nutrient cycling capacity, thereby contributing to improved forage quality (Holatko et al., 2022; Tahir et al., 2023).

Dwarf late (DL) Napier grass is an important C4 tropical forage species widely used in grazing and cut-and-carry systems because of its rapid growth, adaptability, and high biomass production (Jaipolsaen et al., 2022). The species provides substantial amounts of digestible nutrients that contribute to livestock productivity (Habte et al., 2020). Its high photosynthetic efficiency and superior carbon fixation capacity allow greater biomass accumulation than many C3 forage species (Arce Cubas et al., 2023). In addition, DL Napier grass shows good tolerance to heat and drought, making it suitable for cultivation on marginal lands where forage production is often constrained (Pardo and VanBuren, 2021).

Despite its adaptability, maximizing both productivity and nutritive value of DL Napier grass requires adequate N supply because N is essential for chlorophyll formation, leaf development, photosynthesis, and protein synthesis (The et al., 2021). Nitrogen fertilization increases leaf area, chlorophyll concentration, and photosynthetic activity, resulting in greater biomass production (Liu et al., 2023). Since N plays a central role in plant metabolism and energy transfer (Guo et al., 2022), manure can serve as an effective and sustainable nutrient source, particularly under marginal soil conditions where fertility is limited (Luo et al., 2022). While conventional agronomic practices rely heavily on synthetic mineral inputs with literature demonstrating that optimal inorganic N applications can yield between 2,500 and 4,000 g DM m^−2^ annually for tropical panicoid grasses (Galindo et al., 2019; Costa et al., 2020), such chemical intensive approaches are frequently unviable for smallholder systems due to soaring economic costs and supply chain constraints. Consequently, evaluating standalone organic matrices is vital to determine if animal wastes can independently match conventional productivity thresholds.

In addition to biomass production, forage quality is strongly influenced by fiber composition, particularly neutral detergent fiber (NDF), acid detergent fiber (ADF), and acid detergent lignin (ADL). High concentrations of these structural components reduce digestibility and limit nutrient utilization in ruminants (Grześkowiak et al., 2022). Previous studies have shown that manure application can improve forage quality by enhancing nutrient availability and soil biological activity, thereby reducing fiber accumulation and improving digestibility (Mendes et al., 2020; Iqbal et al., 2021). Improved digestibility is important because it directly affects feed intake, nutrient utilization efficiency, and animal performance (Van Soest, 1994; Gaviria-Uribe et al., 2020). Organic fertilization has also been reported to improve soil pH, organic carbon, and nutrient availability, resulting in greater forage productivity in tropical soils (Liu et al., 2010; Du et al., 2020).

Although the beneficial effects of manure on forage production have been widely reported, limited information is available regarding the long-term effects of manure application on both productivity and nutritive value of DL Napier grass under marginal land conditions. Most previous studies mainly focused on short-term biomass responses or inorganic fertilization, while fewer studies simultaneously evaluated forage yield, crude protein concentration, fiber fractions, and digestibility under repeated harvesting systems. Therefore, the novelty of the present study lies in its integrated two-year evaluation of biomass production, forage quality, fiber composition, and digestibility responses of DL Napier grass to different manure application levels in a tropical cut-and-carry system on marginal land. The findings provide practical information for sustainable nutrient management and forage production in integrated crop–livestock systems where access to inorganic fertilizers is limited. To address these critical knowledge gaps, the specific objective of this study was to quantify the longitudinal impacts of increasing composted cattle manure application rates (0.00 to 4.67 g N m^−2^ per application) on the structural yield dynamics, tissue N assimilation, cell-wall lignification kinetics, and ruminal degradability profiles of DL Napier grass over two consecutive production years. We hypothesized that: (i) incremental applications of composted manure would progressively overcome marginal soil constraints to linearize biomass accumulation and N yield without inducing a corresponding increase in recalcitrant fiber fractions (ADF and ADL); and (ii) due to steady OM mineralization over time, the highest manure application level would sustain superior tissue digestibility and structural compliance across successive harvest events in Year 2 compared to Year 1.

## 2 Materials and methods

### 2.1 Experimental site and design

This study was conducted over two years in an experimental field located in Galung Village, Barru District, South Sulawesi Province, Indonesia (**Table 1**). The experimental layout was arranged in a Randomized Complete Block Design with three replications, yielding a total of 12 experimental plots. Each individual plot measured (5 m × 4 m) and served as the primary experimental unit. Dwarf Late (DL) Napier grass (*Cenchrus purpureus*) was established from stem cuttings at a standardized planting density of 2 plants m^−2^ (equivalent to 40 plants per plot). The experiment was managed under strictly rainfed conditions to accurately reflect resource-limited smallholder farming systems. Supplemental water management via manual irrigation was supplied exclusively during the initial establishment period (the first week post-planting only) to guarantee uniform root development, after which the sward relied entirely on natural precipitation. Monthly precipitation and mean temperature data during the two experimental years are presented in **Figure 1**.

**Table 1:** Cultivation and sampling details of dwarf-late Napier grass in 2021 and 2022.

Items	Details	
	2020–2021	2022
Species	Napiergrass (*Cenchrus purpureus*), dwarf-late variety (DL)	
Planting date	November 10, 2020	From regrowth plant
Plant density (plant spacing)	2 plant m^-2^ (50 cm × 100 cm)	
Plot area	20 m^-2^ (5 m × 4 m) plot^-1^	
Manure application level		
Control (C) level	Average 0.00 g N m^-2^ time^-1^)	
Low (L) level	Average 1.38 g N m^-2^ time^-1^	
Medium (M) level	Average 2.88 g N m^-2^ time^-1^	
High (H) level	Average 4.67 g N m^-2^ time^-1^)	
M application date		
1^st^ time	November 16, 2020	January 3
2^nd^ time	February 22	February 20
3^rd^ time	May 10	April 22
4^th^ time	August 26	June 25
5^th^ time	October 10	August 24
Sampling time of soil		
1^st^ time	November 6	January 2
2^nd^ time	February 21	February 19
3^rd^ time	May 9	April 21
4^th^ time	August 25	June 24
5^th^ time	October 9	August 23
Sampling time of plant		
1^st^ time (1st cut)	February 22	February 20
2^nd^ time (2nd cut)	May 10	April 22
3^rd^ time (3rd cut)	August 26	June 25
4^th^ time (4th cut)	October 10	August 24
Measurement of growth characters	Dry matter weight of leaf blade (LB), stem inclusive of leaf sheath (ST) and dead part (D).	
Measurement of manure content	pH, Total Nitrogen (T-N) and Total Phosphoric acid (T-P)	
Measurement of soil content	pH, Total Nitrogen (T-N) and Total Phosphoric acid (T-P)	

**Figure 1:**
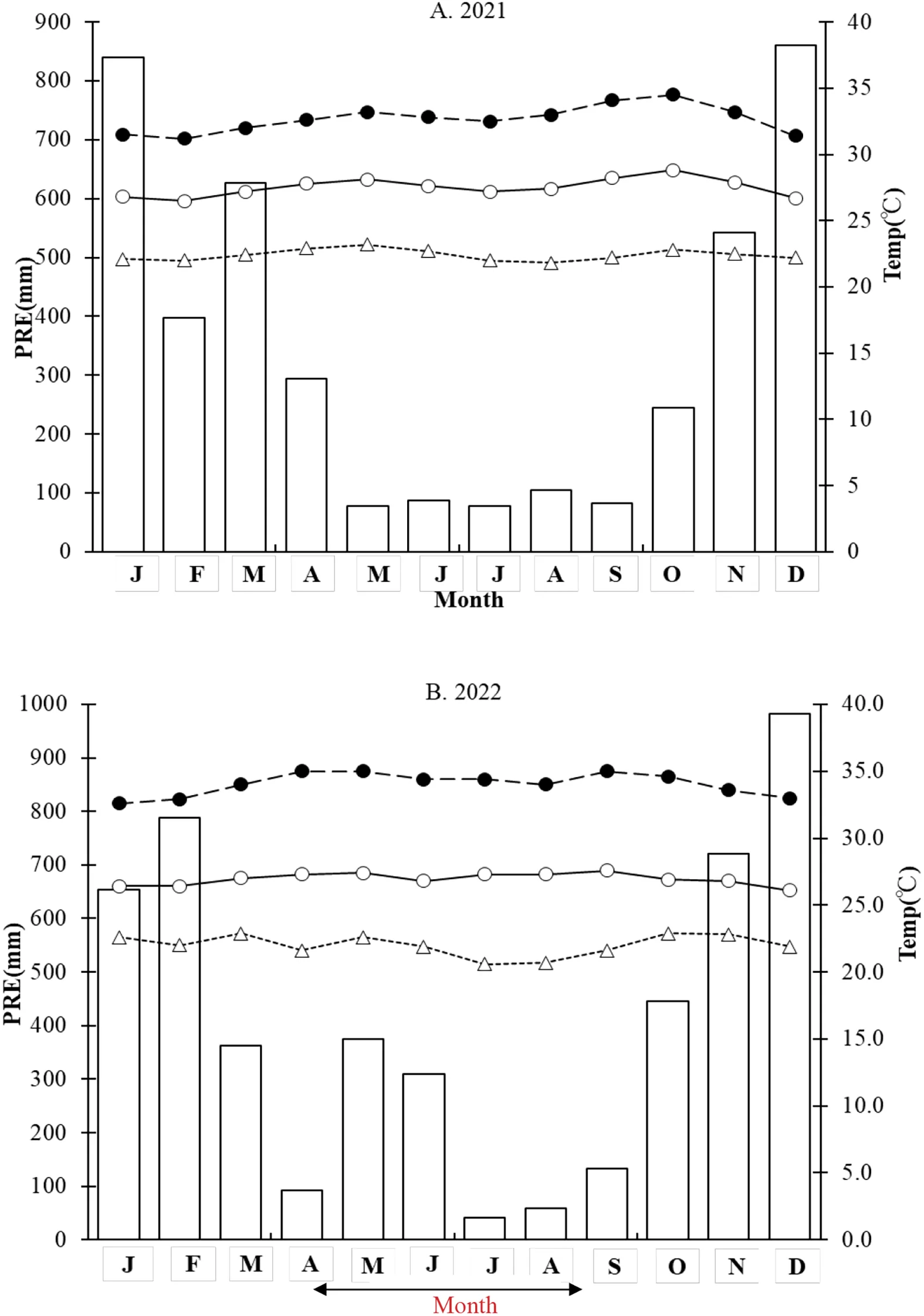
Changes in the mean air temperature (○), maximum air temperature (•), minimum air temperature (△) and precipitation (PRE, □) in the growing season of 2021 **(A)** and 2022 **(B)**.

### 2.2 Manure sampling and physicochemical properties

To monitor temporal variations across the multi-year study, composted cattle manure samples were collected periodically over two experimental years. In the first year (2020–2021), sampling was conducted on November 9, 2020, followed by February 21, May 9, August 24, and October 9, 2021. In the second year (2022), sampling took place on January 2, February 19, April 21, June 24, and August 23. For each experimental year, data collected across the respective sampling dates of manure were compiled to calculate the average annual values for each application level (**Table 2**).

**Table 2:** Physicochemical properties (moisture, total nitrogen, total phosphorus, and pH) of manure during the first (2020–2021) and second (2022) experimental years.

Year	Manure application level	Water content (g 100 g^−1^)	Total nitrogen content (g 100 g^−1^)	Total phosphorus content (g 100 g^−1^)	pH
First	L	26.50 ± 10.82	1.37 ± 0.22	2.68 ± 0.78	9.07 ± 0.15
	M	26.79 ± 10.45	1.44 ± 0.13	2.21 ± 0.86	9.17 ± 0.21
	H	26.59 ± 10.72	1.55 ± 0.26	2.05 ± 0.92	9.21 ± 0.21
Second	L	9.08 ± 0.21	1.58 ± 0.09	2.32 ± 0.19	8.24 ± 0.04
	M	9.06 ± 0.22	1.57 ± 0.11	2.33 ± 0.23	8.25 ± 0.06
	H	9.07 ± 0.21	1.55 ± 0.12	2.28 ± 0.24	8.24 ± 0.08

Manure application of C level (0.00 g N m^-2^ time^-1^), L level (1.38 g N m^-2^ time^-1^), M level (2.88 g N m^-2^ time^-1^), H level (4.67 g N m^-2^ time^-1^). Means and standard deviation of the mean are shown. The complete absence of superscript letters in specific columns explicitly denotes that the main effect of the manure treatment was statistically non-significant (P > 0.05). Values are presented as mean ± standard deviation (SD).

At each sampling interval, manure samples from all treatment levels were evaluated for their physicochemical properties. Moisture content (g 100 g^−1^) and pH were determined alongside chemical composition analyses, which included measuring total nitrogen (T-N, g 100 g^−1^) and total phosphorus (T-P, g 100 g^−1^) contents.

### 2.3 Soil characterization

To evaluate baseline soil fertility and monitor temporal changes in the soil chemical environment, physicochemical characterization (pH, T-N, and T-P) was performed at two key intervals: initially in the first year before the start of the growing period (6 November 2020), and in the second year following the final plant sampling (23 August 2022). The baseline and post-harvest soil characteristics across different manure application levels are presented in **Table 3**.

**Table 3:** Changes in physical properties, macronutrient content (T-N, T-P), and pH of the soil between the first (2020) and second (2022) experimental years.

Year	Manure application level	Water content (g 100 g^−1^)	T-N content (g 100 g^−1^)	T-P content (g 100 g^−1^)	pH
First	C	19.33 ± 3.99	0.132 ± 0.04	2.71 ± 0.76	5.68 ± 0.39
	L	19.32 ± 4.09	0.127 ± 0.04	2.61 ± 0.73	5.92 ± 0.52
	M	19.81 ± 3.95	0.117 ± 0.00	2.21 ± 0.83	6.10 ± 0.56
	H	19.21 ± 4.35	0.108 ± 0.00	2.07 ± 0.88	6.33 ± 0.69
Second	C	4.28 ± 0.19	1.08 ± 0.29	2.79 ± 1.48	5.48 ± 0.28
	L	4.30 ± 0.29	1.06 ± 0.27	2.72 ± 1.49	5.42 ± 0.19
	M	4.20 ± 0.19	1.04 ± 0.30	2.71 ± 1.49	5.65 ± 0.48
	H	4.24 ± 0.27	1.00 ± 0.32	2.69 ± 1.35	5.59 ± 0.52

Manure application of C level (0.00 g N m^-2^ time^-1^), L level (1.38 g N m^-2^ time^-1^), M level (2.88 g N m^-2^ time^-1^), H level (4.67 g N m^-2^ time^-1^). Means and standard deviation of the mean are shown. The complete absence of superscript letters in specific columns explicitly denotes that the main effect of the manure treatment was statistically non-significant (P > 0.05). Values are presented as mean ± standard deviation (SD).

### 2.4 Manure treatments and plant sampling

The organic fertilizer used in this study was composted cattle manure. Manure was applied at four levels: 0.00 (control), 1.38 (low), 2.88 (medium), and 4.67 (high) g N m^−2^ per application. To ensure a consistent nutrient supply, applications were conducted five times during each growing period across 2020-2021 (Nov 16, Feb 22, May 10, Aug, and Oct 10) and 2022 (Jan 3, Feb 20, Apr 22, Jun 25, and Aug 24) (**Table 1**).

Plant sampling was carried out at each defoliation event, with four harvests per year representing successive growth cycles. The first plant samples were collected on February 22, 2021 (105 days after planting). Subsequent harvests followed the defoliation schedule: May 10, August 26, and October 10 for the 2021 growing period, and February 20, April 22, June 25, and August 24 for 2022. To minimize sampling error and avoid border influences, three plants per plot for the measurement of agronomic parameters were selected randomly from the central rows of each plot at each individual harvest event, ensuring edge effects were completely avoided. Different plants were selected at each successive cutting event rather than re-sampling the same individual plants. The sampling process was destructive only to the above-ground biomass at that specific harvest time, as all sampled plants were cut at 10 cm above ground level and immediately allowed to regrow naturally alongside the rest of the plot population for subsequent cycles. Samples were separated into leaf blade (LB), stem with leaf sheath (ST), and dead material. All components were oven-dried at 72 °C to determine dry matter (DM) yield.

### 2.5 Chemical analysis

Total N content of the LB and ST components at each cutting was measured for both years using a CN analyzer (Model NC-220F; Sumigraph). Crude protein content was calculated by multiplying N content by 6.25. *In vitro* dry matter digestibility (IVDMD) of LB and ST was determined using the pepsin–cellulose digestion method in a DAISYII incubator (ANKOM Technology, Macedon, NY, USA) at 39 °C, following the protocol described by Goto and Minson (1977). Briefly, approximately 1 g of dried and ground sample was weighed into F57 filter bags and incubated in the DAISYII incubator at 39 °C for 48 h. After incubation, the bags were removed, washed, dried to constant weight, and weighed. The IVDMD was calculated based on the loss of dry matter during the digestion process. Neutral detergent fiber and ADF were analyzed by detergent assay method with an ANKOM 200 Fiber Analyzer (ANKOM Technology, Macedon, NY, USA) according to the manufacturer’s procedure, while ADL was determined by the sulfuric acid method according to Van Soest et al. (1991). For each sample, all chemical analyses were performed in triplicate, and the mean value of the three replicate analyses was used for statistical analysis. All analyses were conducted consistently across both years.

### 2.6 Statistical analysis

Data were subjected to an analysis of variance (ANOVA) via the GLM procedure of SPSS (version 24). To evaluate the precise temporal behavior of the forage without confounding the distinct structural variations between seasons, and because no repeated-measures or interaction models were analyzed, data from each individual cutting date and experimental year were analyzed separately as independent sward events. This approach prevents Type I error inflation and avoids violating the assumption of independence of residuals. The statistical model for each separate cutting date included the fixed effect of the manure application rate and the random effect of the replication block. Prior to running the ANOVA, the underlying assumptions of linear modeling were verified: data normality was validated using the Shapiro-Wilk test, and homogeneity of variances was confirmed via Levene’s test. Means within each cutting date were compared using Tukey’s test at P < 0.05.

## 3 Results

### 3.1 Yield and nutritive value

Manure application consistently improved both forage yield and nutritive value of DL Napier grass throughout all cutting stages in both years. Dry matter yield (DMY) increased significantly with increasing manure levels, with the highest yields consistently recorded under the high-dose treatment (**Table 4**). Although overall yield was slightly lower in the second year across all application levels, the positive response to manure remained evident, suggesting that nutrient supply and gradual improvement in soil fertility supported plant performance under marginal land conditions. Crude protein (CP) content also increased progressively with manure application, showing a clear dose-dependent response. The highest CP concentrations were observed under the high treatment, whereas the control consistently produced the lowest values.

**Table 4:** Changes in yield and crude protein content in dwarf late Napier grass pasture in the first (1^st^) and second (2^nd^) years.

Year, clipping time, and date	Dry matter yield (g m^-2^)				Crude protein (g 100 g^−1^)			
	Treatment				Treatment			
	C	L	M	H	C	L	M	H
1^st^, I (Feb 22)	125.8 ± 32.30^c^	192.6 ± 77.28^c^	540.2 ± 266.09^b^	1470.0 ± 759.12^a^	8.2 ± 0.11^d^	8.9 ± 0.29^c^	12.3 ± 0.91^b^	14.4 ± 0.25^a^
1^st^, II (May 10)	52.3 ± 23.65^d^	207.0 ± 63.94^c^	376.8 ± 99.81^b^	673.7 ± 210.02^a^	8.5 ± 0.16^d^	9.3 ± 0.12^c^	12.7 ± 0.35^b^	14.4 ± 0.70^a^
1^st^, III (July 26)	111.0 ± 32.88^d^	258.3 ± 62.63^c^	384.2 ± 68.73^b^	752.7 ± 172.58^a^	8.7 ± 0.23^d^	9.5 ± 0.42^c^	13.1 ± 0.81^b^	14.7 ± 0.29^a^
1^st^, IV (Oct 10)	49.9 ± 10.52^c^	107.6 ± 27.62^c^	285.1 ± 7795^b^	479.1 ± 83.31^a^	8.8 ± 0.47^d^	9.9 ± 0.47^c^	13.3 ± 0.29^b^	14.8 ± 0.83^a^
Annual total/mean	339.1 ± 58.65^c^	765.5 ± 89.99^c^	1586.3 ± 221.39^b^	3375.5 ± 665.20^a^	8.5 ± 0.32^d^	9.4 ± 0.46^c^	12.9 ± 0.69^b^	14.6 ± 0.52^a^
2^nd^, I (Feb 20)	118.8 ± 34.79d^d^	257.3 ± 87.93^c^	402.5 ± 109.64^b^	747.3 ± 134.50^a^	7.1 ± 0.26^d^	8.2 ± 0.21^c^	10.2 ± 0.33^b^	12.3 ± 0.74^a^
2^nd^, II (April 22)	55.7 ± 17.10^d^	112.4 ± 25.92^c^	224.4 ± 33.13^b^	364.6 ± 116.44^a^	7.5 ± 0.51^d^	8.7 ± 0.26^c^	10.5 ± 1.23^b^	12.5 ± 0.53^a^
2^nd^, III (Juny 25)	79.3 ± 33.74^d^	185.8 ± 63.43^c^	277.7 ± 72.69^b^	624.1 ± 75.12^a^	7.9 ± 0.40^d^	8.9 ± 0.29^c^	10.8 ± 0.45^b^	12.5 ± 0.27^a^
2^nd^, IV (Aug 24)	49.9 ± 17.10^d^	107.6 ± 25.92^c^	285.1 ± 33.13^b^	479.1 ± 134.50^a^	7.9 ± 0.23^d^	8.7 ± 0.47^c^	10.7 ± 0.60^b^	12.7 ± 0.94^a^
Annual total/mean	303.7 ± 34.83^d^	663.1 ± 102.25^c^	1189.7 ± 156.38^b^	2215.2 ± 299.76^a^	7.6 ± 0.44^d^	8.6 ± 0.38^c^	10.5 ± 0.67^b^	12.6 ± 0.60^a^

Manure application of C level (0.00 g N m^-2^ time^-1^), L level (1.38 g N m^-2^ time^-1^), M level (2.88 g N m^-2^ time^-1^), H level (4.67 g N m^-2^ time^-1^). ^abcd^Means with different superscripts letters in a row denote significant difference for yield or crude protein among treatments on the same date at the 5% level. Values are presented as mean ± standard deviation (SD).

The concentrations of NDF, ADF, and ADL were significantly affected by year, cutting stage, plant part, and manure treatment (**Table 5**). In the first year, leaf blade fiber fractions were generally higher under the control (C) and low (L) manure treatments, while medium (M) and high (H) applications reduced NDF, ADF, and ADL, particularly at later cuttings. Leaf blade NDF ranged from approximately 68.1–73.1 g 100 g^−1^ in C and L treatments but declined under M and H by up to 18.7 g 100 g^−1^ (reaching a minimum of 59.4% in high treatments). A similar trend was observed for ADF, which was lower under M and H (≈39.6–42.4 g 100 g^−1^) compared with C and L (≈43.1–44.0 g 100 g^−1^), reflecting a maximum reduction of 10.0%. ADL was also reduced under higher manure levels, declining by up to 49.4 g 100 g^−1^ from approximately 7.1–8.7 g 100 g^−1^ in C and L to 4.4–6.7 g 100 g^−1^ in M and H treatments.

**Table 5:** Changes in neutral detergent fiber (NDF), acid detergent fiber (ADF) and acid detergent lignin (ADL) contents across cuttings in dwarf Napier grass pasture.

Plant part	Year, clipping time and date	NDF (g 100 g^−1^)				ADF (g 100 g^−1^)				ADL (g 100 g^−1^)			
		C	L	M	H	C	L	M	H	C	L	M	H
Leaf blade	1^st^, I (Feb 22)	71.0 ± 0.21^b^	70.9 ± 0.28^b^	73.1 ± 0.48^a^	69.3 ± 0.31^c^	43.9 ± 0.19^a^	43.7 ± 0.36^a^	41.5 ± 0.42^ab^	40.0 ± 0.77^c^	7.4 ± 0.26^a^	7.1 ± 0.19^a^	5.3 ± 0.32^b^	5.5 ± 0.28^b^
	1^st^, II (May 10)	71.4 ± 0.61^a^	71.2 ± 0.22^a^	63.3 ± 0.11^b^	70.3 ± 1.30^a^	43.7 ± 0.41^a^	43.6 ± 0.58^a^	40.1 ± 0.75^b^	42.4 ± 0.32^a^	7.4 ± 0.61^a^	8.0 ± 0.23^a^	4.4 ± 0.33^b^	4.8 ± 0.21^b^
	1^st^, III (July 26)	70.4 ± 0.74^ab^	68.8 ± 0.55^b^	64.2 ± 0.75^c^	71.4 ± 0.44^a^	44.0 ± 0.64^a^	43.7 ± 0.50^a^	39.6 ± 0.41^b^	43.6 ± 0.33^a^	7.4 ± 0.39^a^	7.4 ± 0.41^a^	5.4 ± 0.28^b^	4.6 ± 0.49^b^
	1^st^, IV (Oct 10)	69.7 ± 0.28^a^	68.1 ± 0.94^b^	66.7 ± 0.20^c^	65.1 ± 0.02^d^	43.4 ± 0.37^a^	43.1 ± 0.20^a^	41.7 ± 0.83^ab^	40.1 ± 0.32^c^	8.7 ± 0.33^a^	8.1 ± 0.94^ab^	6.7 ± 0.23b	5.1 ± 0.32c
	Annual total/mean	70.6 ± 0.80^a^	69.7 ± 1.46^a^	66.8 ± 4.03^b^	69.0 ± 2.56^ab^	43.8 ± 0.45^a^	43.5 ± 0.44^a^	40.7 ± 1.09^b^	41.5 ± 1.66^b^	7.7 ± 0.70^a^	7.7 ± 0.63^a^	5.5 ± 0.90^b^	5.0 ± 0.47^b^
Stem with leaf sheath	1^st^, I (Feb 22)	71.9 ± 0.86^a^	71.4 ± 0.27^a^	66.7 ± 0.31^bc^	69.5 ± 0.15^ab^	47.7 ± 0.40^a^	47.6 ± 0.50^a^	44.3 ± 1.01^b^	45.3 ± 0.21^b^	7.7 ± 0.15^b^	9.6 ± 0.62^a^	5.5 ± 0.51^c^	7.4 ± 0.53^b^
	1^st^, II (May 10)	70.3 ± 0.32^b^	69.9 ± 0.41^b^	68.0 ± 0.46^c^	72.6 ± 0.54^a^	44.3 ± 0.26^a^	44.2 ± 0.21^a^	41.8 ± 0.41^b^	41.3 ± 0.70^b^	7.3 ± 0.32^b^	9.4 ± 0.33^a^	7.7 ± 0.32^b^	7.2 ± 0.16^b^
	1^st^, III (July 26)	71.1 ± 0.47^a^	70.8 ± 0.79^a^	70.6 ± 0.34^a^	66.2 ± 0.26^b^	45.9 ± 0.59^a^	45.4 ± 0.42^a^	42.3 ± 0.32^b^	41.3 ± 0.24^b^	7.1 ± 0.16^b^	8.9 ± 0.31^a^	6.7 ± 0.32^bc^	6.0 ± 0.22c
	1^st^, IV (Oct 10)	70.9 ± 0.90^a^	69.5 ± 0.45^a^	67.5 ± 0.51^ab^	66.1 ± 0.12^b^	43.9 ± 0.37^a^	43.5 ± 0.45a	41.5 ± 0.44^b^	41.1 ± 1.05^b^	7.9 ± 0.47^a^	8.5 ± 0.45^a^	6.5 ± 0.39^b^	6.1 ± 0.12^b^
	Annual total/mean	71.0 ± 0.83^a^	70.4 ± 0.91^ab^	68.2 ± 1.58^c^	68.6 ± 2.82^bc^	45.4 ± 1.60^a^	45.2 ± 1.68^a^	42.5 ± 1.24^b^	42.3 ± 1.92^b^	7.5 ± 0.43^b^	9.1 ± 0.60^a^	6.6 ± 0.86^c^	6.7 ± 0.70^c^
Leaf blade	2^nd^, I (Feb 20)	64.1 ± 0.06	62.9 ± 0.86	63.6 ± 0.31	64.0 ± 0.38	39.3 ± 0.26^b^	40.1 ± 0.51^ab^	40.3 ± 0.45^ab^	40.7 ± 0.32^a^	10.5 ± 0.45	10.1 ± 0.10	9.5 ± 0.43	10.5 ± 0.43
	2^nd^, II (April 22)	66.1 ± 0.51^a^	66.0 ± 0.70a	66.5 ± 0.41a	64.6 ± 0.19b	41.1 ± 0.60^b^	42.6 ± 0.29^a^	42.7 ± 0.41^a^	43.8 ± 0.82^a^	10.9 ± 0.40a^b^	11.7 ± 0.66^a^	10.2 ± 0.37^b^	10.4 ± 0.52^b^
	2^nd^, III (June 25)	61.9 ± 0.55	62.2 ± 0.11	61.8 ± 0.28	61.7 ± 0.75	39.9 ± 0.57^c^	40.7 ± 0.31^abc^	40.9 ± 0.81^abc^	41.5 ± 0.17^a^	8.9 ± 0.41^c^	11.9 ± 0.55^a^	10.2 ± 0.17^b^	9.8 ± 0.20^bc^
	2^nd^, IV (Aug 24)	62.1 ± 0.14^a^	61.5 ± 0.38a^ab^	58.3 ± 0.57^c^	59.5 ± 0.18^b^	38.6 ± 0.29^c^	39.7 ± 0.56^b^	40.2 ± 0.31^ab^	41.0 ± 0.45^a^	11.9 ± 0.64^a^	11.4 ± 0.57^ab^	10.9 ± 0.28^ab^	10.6 ± 0.36^b^
	Annual total/mean	63.5 ± 1.80	63.2 ± 1.88	62.5 ± 3.10	62.4 ± 2.17	39.7 ± 1.04b	40.8 ± 1.22^ab^	41.0 ± 1.14^a^	41.7 ± 1.33^a^	10.5 ± 1.20^ab^	11.3 ± 0.84^a^	10.2 ± 0.56^b^	10.3 ± 0.46^b^
Stem with leaf sheath	2^nd^, I (Feb 20)	65.1 ± 0.19^a^	63.4 ± 0.08^bc^	62.7 ± 0.36^c^	64.0 ± 0.46^b^	37.9 ± 0.31	38.4 ± 0.27	38.4 ± 0.37	38.7 ± 0.32	10.7 ± 0.49^ab^	11.8 ± 0.64^a^	10.2 ± 0.14^b^	8.3 ± 0.26^c^
	2^nd^, II (April 22)	65.5 ± 0.46^a^	62.5 ± 0.32^b^	63.3 ± 0.39^b^	62.7 ± 0.65^b^	40.1 ± 0.36^c^	40.6 ± 0.58^abc^	41.1 ± 0.55^abc^	41.8 ± 0.29^a^	7.6 ± 0.47^b^	8.4 ± 0.54^ab^	8.8 ± 0.26^a^	7.7 ± 0.40^ab^
	2^nd^, III (June 25)	63.8 ± 0.21^a^	63.7 ± 0.47^a^	62.9 ± 0.07^b^	64.4 ± 0.35^a^	40.6 ± 0.56^c^	41.3 ± 0.39^bc^	42.3 ± 0.45^ab^	42.8 ± 0.56^aa^	7.9 ± 0.25	8.7 ± 0.74	9.3 ± 0.46	8.5 ± 0.53
	2^nd^, IV (Aug 24)	62.1 ± 0.04^b^	62.8 ± 0.48^c^	62.6 ± 0.03^bc^	63.5 ± 0.07^a^	38.9 ± 0.46^b^	39.8 ± 0.29^ab^	39.9 ± 0.54^ab^	40.5 ± 0.38^a^	9.2 ± 0.10^a^	8.5 ± 0.35^ab^	7.8 ± 0.15^b^	7.8 ± 0.51^b^
	Annual total/mean	64.1 ± 1.40^a^	63.1 ± 0.60^b^	62.9 ± 0.36^b^	63.6 ± 0.75^ab^	39.4 ± 1.16^c^	40.0 ± 1.18^abc^	40.4 ± 1.56^abc^	40.9 ± 1.61^a^	8.9 ± 1.31^ab^	9.4 ± 1.53^a^	9.0 ± 0.94^ab^	8.1 ± 0.51^b^

Manure application of C level (0.00 g N m^-2^ time^-1^), L level (1.38 g N m^-2^ time^-1^), M level (2.88 g N m^-2^ time^-1^), H level (4.67 g N m^-2^ time^-1^). Different letters in a row denote significant difference for NDF, ADF and ADL among treatments on the same date at the 5% level. The complete absence of superscript letters in specific rows explicitly denotes that the main effect of the manure treatment was statistically non-significant (P > 0.05). Values are presented as mean ± standard deviation (SD).

In stem with leaf sheath tissues, NDF remained relatively high (≈66.1–72.6 g 100 g^−1^), although slight reductions were observed under the high treatment. ADF values were higher in C and L (≈43.5–47.7 g 100 g^−1^) and lower in M and H (≈41.1–45.3 g 100 g^−1^), demonstrating a decrease of up to 13.8 g 100 g^−1^ under heavy inputs. The highest ADL concentration was recorded under the low treatment, while medium and high manure applications generally reduced lignin content by up to 86.2 g 100 g^−1^ relative to the low treatment baseline.

During the second year, overall fiber concentrations were lower than in the first year. Leaf blade NDF ranged from 58.3 to 66.5 g 100 g^−1^, while ADF varied from 38.6 to 43.8 g 100 g^−1^, with treatment effects becoming less pronounced. However, ADL remained comparatively high, especially under the low treatment (≈8.9–11.9 g 100 g^−1^). Stem tissues showed relatively stable NDF (≈62.1–65.5 g 100 g^−1^) and ADF (≈38.9–42.8 g 100 g^−1^) values, whereas ADL ranged from 7.6 to 11.8 g 100 g^−1^. Across both years, stems consistently contained higher fiber fractions than leaves. Overall, medium and high manure treatments reduced NDF, ADF, and ADL concentrations more effectively than control and low treatments, with the clearest effects observed during the first year.

### 3.2 *In vitro* dry matter digestibility and correlation analysis

Manure application had limited effects on IVDMD during the first year, with significant differences observed only in some clippings of the stem with leaf sheath fraction (**Table 6**). For example, first-year leaf blade IVDMD remained statistically unchanged during Cutting I, hovering between 64.91 g 100 g^−1^ and 67.06 g 100 g^−1^ across all swards. In the second year, however, digestibility increased with manure application rate. The medium (M) and high (H) manure treatments generally produced higher IVDMD values than the control (C) and low (L) treatments, particularly in the third and fourth clippings. Notably, during Cutting III of Year 2, stem IVDMD rose by 6.06 absolute percentage points, climbing from 71.87 g 100 g^−1^ in control plots to 77.93 g 100 g^−1^ at the highest application rate. Similarly, combined whole-plant (leaf + stem) IVDMD during Cutting III improved by 5.44 absolute percentage points (rising from 71.62 g 100 g^−1^ in controls to 77.06 g 100 g^−1^ under high application rates). Overall, higher manure application improved forage digestibility, indicating enhanced feeding value of dwarf Napier grass.

**Table 6:** Changes in *in vitro* dry matter digestibility (g 100 g^−1^) across cuttings in dwarf Napier grass pasture.

Plant part	Year, clipping time and date	C	L	M	H
Leaf blade	1^st^, I (Feb 22)	64.9 ± 0.79	65.7 ± 0.17	66.9 ± 1.46	67.0 ± 0.84
	1^st^, II (May 10)	64.6 ± 0.06^c^	66.2 ± 0.70^b^	67.1 ± 0.44^ab^	68.1 ± 0.10^a^
	1^st^, III (July 26)	72.3 ± 0.21^a^	71.7 ± 0.08^b^	68.9 ± 0.06^c^	68.7 ± 0.14^c^
	1^st^, IV (Oct 10)	68.5 ± 0.09^c^	68.6 ± 0.05^c^	69.2 ± 0.09^b^	69.8 ± 0.12^a^
Stem with leaf sheath	1^st^, I (Feb 22)	70.6 ± 0.07^c^	72.1 ± 0.25^b^	72.9 ± 0.06^a^	73.2 ± 0.04^a^
	1^st^, II (May 10)	69.0 ± 0.05^d^	69.2 ± 0.03^c^	70.8 ± 0.02^b^	73.6 ± 0.07^a^
	1^st^, III (July 26)	74.9 ± 0.38^a^	73.9 ± 0.09^b^	70.7 ± 0.20^c^	70.5 ± 0.21^c^
	1^st^, IV (Oct 10)	69.9 ± 0.10	69.6 ± 0.04	69.5 ± 0.09	69.5 ± 0.44
Leaf blade + stem with leaf sheath	1^st^, I (Feb 22)	67.0 ± 0.17^a^	68.0 ± 0.81^ab^	69.2 ± 0.08^bc^	70.2 ± 0.85^c^
	1^st^, II (May 10)	65.7 ± 0.45^d^	67.2 ± 0.12^c^	68.6 ± 0.47^b^	70.6 ± 0.54^a^
	1^st^, III (July 26)	73.0 ± 0.16^a^	72.3 ± 0.29^a^	69.4 ± 0.57^b^	69.3 ± 0.47^b^
	1^st^, IV (Oct 10)	68.8 ± 0.78	68.8 ± 0.99	69.3 ± 0.36	69.7 ± 0.21
Leaf blade	2^nd^, I (Feb 20)	69.8 ± 0.46	70.0 ± 0.82	70.6 ± 1.10	71.3 ± 0.44
	2^nd^, II (April 22)	76.1 ± 0.94	76.4 ± 1.01	77.2 ± 0.42	77.4 ± 0.53
	2^nd^, III (June 25)	71.6 ± 1.40^b^	74.3 ± 0.98a^b^	76.2 ± 0.63^a^	76.6 ± 1.23^a^
	2^nd^, IV (Aug 24)	74.1 ± 0.91^b^	74.5 ± 0.89^b^	76.6 ± 0.50^a^	76.7 ± 0.69^a^
Stem with leaf sheath	2^nd^, I (Feb 20)	70.00 ± 0.34^b^	70.21 ± 0.20^b^	72.44 ± 1.33^ab^	73.59 ± 1.53^a^
	2^nd^, II (April 22)	76.4 ± 1.22^b^	76.7 ± 1.02^b^	79.2 ± 0.66^a^	78.8 ± 1.17^ab^
	2^nd^, III (June 25)	71.8 ± 2.00^b^	74.5 ± 0.50^b^	77.7 ± 1.09^a^	77.9 ± 0.06^a^
	2^nd^, IV (Aug 24)	74.4 ± 1.28^b^	74.9 ± 1.04^b^	77.9 ± 0.13^a^	78.1 ± 1.46^a^
Leaf blade + stem with leaf sheath	2^nd^, I (Feb 20)	69.8 ± 0.76^a^	70.1 ± 0.25^ab^	71.3 ± 0.43^bc^	72.2 ± 0.17^c^
	2^nd^, II (April 22)	76.1 ± 0.10^a^	76.5 ± 0.51^a^	77.7 ± 0.40^b^	77.8 ± 0.32^b^
	2^nd^, III (June 25)	71.6 ± 0.32^a^	74.3 ± 0.34^b^	76.5 ± 0.45^c^	77.0 ± 0.16^c^
	2^nd^, IV (Aug 24)	74.2 ± 0.10^a^	74.6 ± 0.37^a^	76.9 ± 0.41^b^	77.0 ± 0.18^b^

Manure application of C level (0.00 g N m^-2^ time^-1^), L level (1.38 g N m^-2^ time^-1^), M level (2.88 g N m^-2^ time^-1^), H level (4.67 g N m^-2^ time^-1^). Different letters in a row denote significant difference among treatments on the same date at the 5% level. Values are presented as mean ± standard deviation (SD). The complete absence of superscript letters in specific rows (including all four cuttings of the Leaf Blade in the first year, and the combined Leaf Blade + Stem fraction where indicated) explicitly denotes that the main effect of the manure treatment was statistically non-significant (P > 0.05).

Physiological assessments across independent harvest events confirmed that cell wall accumulation was a primary factor limiting forage utilization in DL Napier grass swards. Across individual growth intervals, drops in whole-plant IVDMD tracked closely with rapid structural cell-wall loading, meaning higher tissue maturity and NDF concentration drove structural changes in forage degradation. Furthermore, canopy growth dynamics highlighted that changes in DMY closely followed improvements in structural agronomic traits. Increases in biomass were physically mediated by enhanced plant height, expanded leaf area index (LAI), and accelerated crop growth rates (CGR), whereas absolute accumulation values decreased when the sward developed a higher proportion of senescent or dead material.

## 4 Discussion

The results demonstrate that manure application significantly enhanced both biomass production and forage quality of DL Napier grass over the two-year period. The steady increase in DM yield with higher manure levels indicates that N supplied through manure was a key driver of plant growth. It is hypothesized that improved N availability likely promoted leaf expansion, canopy development, and metabolic activity, leading to greater biomass accumulation. Nitrogen is an essential component of chlorophyll, amino acids, proteins, and several enzymes involved in plant growth and photosynthesis; therefore, an adequate N supply can promote vegetative development and biomass accumulation (The et al., 2021; Costa et al., 2020). Previous studies have also demonstrated positive effects of manure application on crop productivity, with meta-analysis evidence showing that manure fertilizer can improve crop yield and soil properties (Du et al., 2020). Similar positive responses of forage production to organic fertilization have been reported in forage systems (Hays et al., 2023; Villa-Galaviz et al., 2023).

The consistent response across both years suggests that manure not only supplied nutrients but also may have contributed to gradual improvements in soil conditions. Organic amendments are known to enhance soil structure, microbial activity, water retention, and nutrient buffering capacity, which are particularly important in marginal land systems. For example, long-term manure application has been associated with improvements in soil chemical and biological properties and crop productivity (Liu et al., 2010; Loss et al., 2022), while cattle manure amended with biochar and humic substances has been reported to improve soil microbiological properties and nutrient status (Holatko et al., 2022). In addition, manure-derived N is released progressively through mineralization, providing a potential source of plant-available N over time (Singh et al., 2020). However, as these specific soil physical and biological parameters were not directly measured in the present study, they remain inferred mechanisms that require future experimental validation. These cumulative effects likely supported sustained forage productivity throughout the experimental period.

Although the trend of increasing yield with manure application was maintained, a slight reduction in DM yield was observed in the second year. This may be attributed to seasonal variability and progressive nutrient depletion under continuous harvesting, where nutrient removal through repeated cuttings may have exceeded the immediate mineralization rate of organic inputs. Napier grass is a high-yielding perennial forage and its repeated harvesting requires an adequate and continuous supply of nutrients to maintain biomass production (Habte et al., 2020; Costa et al., 2020). Intriguingly, this second-year yield decline coincided with a substantial improvement in overall forage digestibility, particularly within the stem fraction, presenting a minor physiological paradox. This interannual dynamic can be explained morphologically by a notable shift in the plant’s structural biomass allocation: under the lower overall growth rates of the second year, the dwarf late Napier grass allocated proportionally more harvested biomass to the leaf blade fraction (averaging a higher leaf-to-stem ratio across cutting dates) relative to the more fibrous stem tissue. Because LB inherently possess a significantly higher nutritive value than ST, this altered morphological composition effectively elevated mean whole-plant IVDMD despite the drop in absolute dry matter accumulation. The relationship between plant morphology, maturity, fiber concentration, and digestibility is well established in forage crops, with increased structural tissue development generally associated with greater cell-wall concentration and lower digestibility (Van Soest, 1994). This process was potentially supplemented by a soil microbial priming effect; however, because soil microbial activity and N mineralization were not directly measured in the present study, this mechanism should be regarded as a possible explanation rather than a confirmed effect. Nevertheless, the overall positive response confirms the importance of continued nutrient management in long-term cut-and-carry systems.

Crude protein content increased with higher manure application, reflecting the direct role of N in protein synthesis. The strong positive association between DM yield and CP further indicates that improved N availability enhanced both productivity and nutritive value simultaneously. Since N in manure undergoes microbial mineralization before plant uptake, the observed increase in CP also suggests active soil biological processes contributing to nutrient cycling (Singh et al., 2020; Chauhan et al., 2021; Oliveira et al., 2022). This finding is consistent with previous research showing that increasing N availability enhances both DM and protein accumulation in forage grasses (Oliveira et al., 2022). Similarly, application of cow-dung biodigester slurry has been shown to influence the dry matter and crude protein characteristics of Napier grass (Rambau et al., 2022). These findings support the interpretation that manure-derived N contributed to the increased CP concentration observed in the present study.

In contrast, CP showed little association with IVDMD, indicating that protein content alone is not a reliable predictor of forage digestibility. Instead, digestibility was more closely related to fiber composition. The NDF, ADF, and ADL generally decreased with increasing manure application, particularly in the first year. This reduction suggests that manure improved plant tissue characteristics by reducing structural rigidity and lignification, resulting in more digestible forage. The importance of cell-wall components in determining forage digestibility has been extensively established, with NDF, ADF, and lignin being important indicators of the structural fraction of forage and its potential utilization by ruminants (Van Soest et al., 1991; Van Soest, 1994). Previous research has also shown that manure-based fertilization can influence forage nutritional characteristics. For example, Walie et al. (2022) reported differences in the nutritional value and *in vitro* fermentation characteristics of forage grasses cultivated with farmyard manure. Rambau et al. (2022) also demonstrated that fertilization with cow-dung biodigester slurry affected the nutritional characteristics and degradability of Napier grass. Similar improvements in forage quality through organic fertilization have been reported in other forage systems (Loss et al., 2022; Villa-Galaviz et al., 2023).

Mechanistically, high N availability from organic matrices stimulates rapid meristematic activity and leaf elongation. This vigorous vegetative growth accelerates the synthesis and accumulation of highly digestible soluble cellular contents, cellulose, and hemicellulose relative to total cell wall structural deposition. Consequently, the expanding canopy maintains a higher leaf-to-stem ratio. Across successive harvests, this delayed maturity shifts the accumulation patterns of cell wall fractions away from heavy NDF loading and aggressive tissue lignification (ADL accumulation), generating younger, structurally compliant tillers that optimize whole-plant IVDMD. Previous studies have demonstrated that N availability influences vegetative growth, biomass accumulation, and forage nutritional characteristics (Costa et al., 2020; Oliveira et al., 2022). However, because leaf-to-stem ratio, plant maturity, and cell-wall deposition were not directly measured as causal mechanisms in the present study, these mechanisms should be considered possible explanations rather than definitive conclusions.

These quality shifts align closely with observations of intensive tropical forage management across other regions. Organic manure has been reported to improve forage production and soil fertility in a range of forage-based systems. Villa-Galaviz et al. (2023), for example, demonstrated that manure fertilization could support forage production while reducing some of the trade-offs associated with intensive grassland management. Similarly, Walie et al. (2022) reported that farmyard manure management influenced the nutritional value of forage grasses. These findings indicate that organic nutrient sources can contribute to both forage productivity and quality, although responses may vary according to soil conditions, manure characteristics, application rate, and forage species.

However, smallholder implementation faces key practical limitations. Manure composition is notoriously variable depending on livestock diet and storage conditions, making precise N delivery unpredictable. Furthermore, manual cut-and-carry operations carry steep logistical constraints and labor costs, alongside potential biological contamination risks if the manure matrix is not fully composted. Economically, the collection, hauling, and manual surface broadcasting of high bulk volumes present clear resource trade-offs for smallholders. The nutrient content and fertilizer value of manure can vary considerably with its source, handling, storage, and processing, which may complicate the determination of precise nutrient application rates. Therefore, manure application based on nutrient content rather than only application weight may improve nutrient-use efficiency (Du et al., 2020; Loss et al., 2022).

From an environmental standpoint, utilizing manure on marginal soils facilitates circular nutrient cycling and offers a viable pathway to reduce heavy reliance on synthetic chemical fertilizers. Nonetheless, this organic management introduces environmental trade-offs. Manure recycling can contribute to nutrient recovery and reduce dependence on external fertilizer inputs; however, inefficient nutrient management can also increase the risk of nutrient losses to the environment. Studies of integrated crop–livestock systems have emphasized the importance of managing N inputs and losses to improve nutrient-use efficiency (Du et al., 2022). The surface application of heavy organic matter loads may create conditions that influence greenhouse gas emissions, notably nitrous oxide (N_2_O) through nitrification-denitrification pathways and methane (CH_4_) under wet conditions. However, greenhouse gas emissions were not measured in the present study, and therefore these potential effects should not be interpreted as direct findings. Future long-term work must measure trace gas fluxes alongside agronomic yields to balance smallholder productivity with global sustainability goals.

Year-to-year variations in the relationship between fiber components and DM yield suggest shifts in plant structural allocation over time. In the first year, higher fiber content was associated with greater structural development, whereas in the second year, improved soil conditions may have supported higher biomass production with relatively lower fiber accumulation, indicating more efficient growth dynamics. Such variation may result from differences in environmental conditions, plant maturity, cutting interval, nutrient availability, and soil nutrient status. Previous studies have demonstrated that nutrient management can influence both biomass accumulation and forage chemical composition (Costa et al., 2020; Oliveira et al., 2022). Therefore, the differences observed between years in the present study may reflect the combined effects of manure application and year-specific growing conditions.

Overall, manure application influenced not only nutrient supply but also plant structural development and forage quality. The simultaneous improvement in DM yield, CP content, and digestibility, along with reduced fiber fractions, demonstrates the dual benefit of manure as both a fertilizer and a forage quality enhancer. These effects are closely linked to inferred changes in soil fertility and enhanced microbial activity, which contribute to system stability over time (Villa-Galaviz et al., 2023; Walie et al., 2022). Nevertheless, because soil fertility changes and microbial activity were not directly measured, these mechanisms should be interpreted cautiously. The strongest evidence from the present study is the measured improvement in DM yield and CP concentration, together with the changes observed in IVDMD, NDF, ADF, and ADL.

These findings are particularly relevant for smallholder livestock systems in tropical regions where access to inorganic fertilizers is limited. Manure-based nutrient management supports circular nutrient recycling between livestock and forage production systems, reducing external input dependence while improving forage productivity and quality. The recycling of livestock manure into forage production can return nutrients to the soil and potentially reduce dependence on synthetic fertilizers, while simultaneously providing a productive use for livestock waste (Du et al., 2020, 2022). Overall, the study provides practical evidence for sustainable intensification of forage production on marginal lands.

## 5 Conclusions

This two-year study demonstrated that manure application significantly influenced consistently improved biomass production, nutritive value, and digestibility dynamics of DL Napier grass under rainfed marginal land conditions. Higher manure levels resulted in increased DM yield, and enhanced CP content, while contributing to a reduction in structural fiber fractions and localized increases in tissue digestibility during individual harvest cycles. Sward structural screening indicated that growth traits closely tracked absolute biomass production, while fiber components coincided with changes in cell wall rigidity and degradation traits. Among the treatments, the highest manure level (4.67 g N m^−2^ per application) exhibited the highest capacity to alter yield and quality parameters under the constraints of natural precipitation. This level shows strong potential for DL Napier grass grown under a cut-and-carry system on marginal land, offering a standalone organic alternative to chemical fertilization inputs. While further long-term monitoring is required to validate the exact impacts on soil physical structures and greenhouse gas trace fluxes, these short-term agronomic outcomes highlight the potential value of manure-based nutrient management as a sustainable approach for improving forage production in tropical integrated crop–livestock systems.

## Data Availability

The raw data supporting the conclusions of this article will be made available by the authors, without undue reservation.
